# Tunable structural and optical properties of CuInS_2_ colloidal quantum dots as photovoltaic absorbers†

**DOI:** 10.1039/d1ra03659a

**Published:** 2021-06-16

**Authors:** Shanna-Kay Ming, Richard A. Taylor, Paul D. McNaughter, David J. Lewis, Marina A. Leontiadou, Paul O'Brien

**Affiliations:** Department of Chemistry, University of the West Indies St. Augustine Trinidad and Tobago richard.taylor@sta.uwi.edu; Department of Chemistry, University of Manchester M13 BB UK; Department of Materials, University of Manchester Manchester M13 9PL UK; Department of Physics and Astronomy & Photon Science Institute, The University of Manchester Manchester M13 9PL UK

## Abstract

Facile phase selective synthesis of CuInS_2_ (CIS) nanostructures has been an important pursuit because of the opportunity for tunable optical properties of the phases, and in this respect is investigated by hot-injection colloidal synthesis in this study. Relatively monodispersed colloidal quantum dots (3.8–5.6 nm) of predominantly chalcopyrite structure synthesized at 140, 180 and 210 °C over 60 minutes from copper(ii) hexafluoroacetylacetonate hydrate and indium(iii) diethyldithiocarbamate precursors exhibit temperature-dependent structural variability. The slightly off-stoichiometric quantum dots are copper-deficient in which copper vacancies 
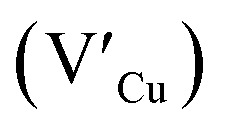
, indium interstitials 
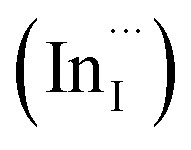
, indium–copper anti-sites 
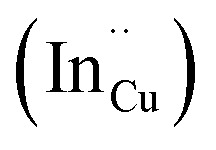
 and surface trapping states are likely implicated in broad photoluminescence emission with short radiative lifetimes, *τ*_1_, *τ*_2_, and *τ*_3_ of 1.5–2.1, 7.8–13.9 and 55.2–70.8 ns and particle-size dependent tunable band gaps between 2.25 and 2.32 eV. Further structural and optical tunability (*E*_g_ between 2.03 and 2.28 eV) is achieved with possible time-dependent wurtzite to chalcopyrite phase transformation at 180 °C likely involving a dynamic interplay of kinetic and thermodynamic factors.

## Abstract 2

We report the facile hot-injection colloidal synthesis of near-stoichiometric CuInS_2_ quantum dots at varying reaction times and temperatures which exhibit both optical and structural tunability with implications for enhanced photovoltaic utility.

## Graphical abstract

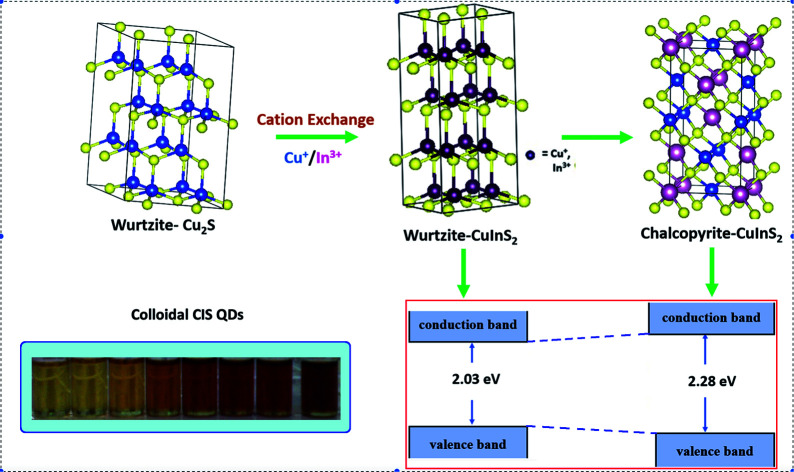

## Introduction

Multinary copper chalcogenide nanocrystals (NCs) such as copper indium sulphide (CuInS_2_) are an extraordinarily interesting class of materials. This is because of their (i) excellent intrinsic functional properties, including direct band gaps appropriate for solar energy utility, remarkable charge carrier mobilities and densities, (ii) versatility of structure, composition, and stoichiometry, including large off-stoichiometry and abundant non-stoichiometric phases and (iii) earth abundance, low cost, environmental and benign health effects compared to cadmium and lead-based counterparts.^1–5^ Additionally, metal chalcogenide NCs smaller than twice the Bohr exciton radius, *i.e.* quantum dots (QDs), typically exhibit a unique combination of high optical absorptivity, photoluminescence with high quantum yields (QYs) and strong particle size-dependent band gap tunability.^6^ These, in combination allow for broad variation of photophysical properties attractive for more spectrally adaptive thin film and quantum dot solar cells.

As the more thermodynamically stable phase, the chalcopyrite (CH) structure of CIS has been mostly studied because of its tolerance for a large range of anion and cation off-stoichiometry which allows doping defects of either p- or n-type conductivity, tunable band gap and photoluminescence properties. These tunable properties are influenced by the presence of intrinsic defects such as sulphur vacancies 
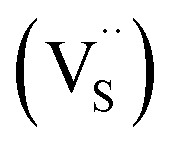
 and interstitials 
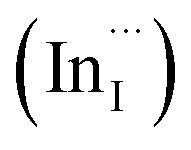
 as donors and copper vacancies 
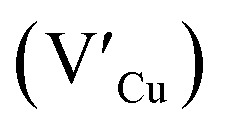
 as acceptors; types implicated in the off-stoichiometric phases. In addition to the chalcopyrite (CH) phase that is stable up to 980 °C, the phase diagram of the Cu_2_S–In_2_S_3_ ternary system suggests that CuInS_2_ exists in two other crystal phases: (i) zinc blende (ZB) between 980 and 1045 °C and (ii) wurtzite (WZ) between 1045 and 1090 °C, with all three stable in nanocrystals at room temperature.^1,7^ In the bulk state, wurtzite CuInS_2_ with band gap near the red edge of the visible spectrum is less investigated with some recent reports highlighting the mechanism of formation and optical properties,^8^ including for various nanocrystals.^9^ Along with high optical absorption coefficients and substantial photostability, WZ phase CIS is equally promising as a light-absorbing material in printed and flexible photovoltaic devices, light-emitting diodes and nonlinear optical devices.^9^

Notwithstanding the attractive properties of CIS, the highest recorded power conversion efficiencies (PCEs) in solar cells are not as competitive as silicon-based cells with a PCE of 26.5%.^10^ Efficiencies for quantum dot based cells are about 7–8% with thin film cells at 13%.^11–13^ Accordingly, the facile and predictable tunability of structural and optoelectronic properties of CIS is a critical means through which PCEs can be improved especially by manipulating intrinsic and extrinsic defect chemistry. Though CIS NCs have been studied by several research groups, part of the challenge has been the need for optimised synthetic protocols to easily and effectively control composition, stoichiometry, structure and particle size, and these efforts continue to be worthwhile pursuits within the scope of semiconductor materials engineering. More notable is the paucity of reports on WZ structure CIS which shows opportunity for further investigations. From that perspective, herein reported are structural and optical properties of fairly monodispersed colloidal CIS QDs synthesized *via* hot injection involving copper(ii) hexafluoroacetylacetonate hydrate and indium(iii) diethyldithiocarbamate precursors at moderate temperatures. A formation mechanism based on the reactivity and decomposition of precursors is proposed considering the interplay of kinetic and thermodynamic factors. Additionally, the influence of composition, stoichiometry and defect chemistry on the structural and optical properties is discussed in reference to synthetic variables. Importantly, time-dependent wurtzite to chalcopyrite phase transformation during growth at constant temperature is investigated as a unique alternative route towards tunable structural and optical properties.

## Results and discussion

### Synthetic route

In this synthetic scheme, formation of CIS colloidal nanoparticles can occur *via* cation exchange of In^3+^ with Cu^+^ of copper sulphide nanoparticles *in situ* or thermolysis of *in situ* precursor Cu–In thiolate complex,^17^ the former more likely since dodecanethiol (DDT) capping ligand, a soft base would preferentially react with (and reduce) Cu^2+^ ion, a soft Lewis acid, than with In^3+^ ion, a hard Lewis acid. Since the copper precursor decomposes initially during the colloidal mechanism (see Fig. S1 ESI†), a higher concentration of Cu^+^ ions than In^3+^ ions at this stage would propel the condition for copper sulphide (CuS/Cu_2−*x*_S) nucleation. The unstable nanoparticles then undergo cation exchange with In^3+^ ions forming stable DDT-capped CIS nanocrystals. As an alternative, the presence of oleylamine (OLA) in the DDT mixture would increase the reactivity of the In^3+^ ions propelling the formation of Cu–In thiolate with subsequent thermolysis precipitating colloidal CIS QDs.^8,18^

### Structural elucidation

**Fig. 1:**
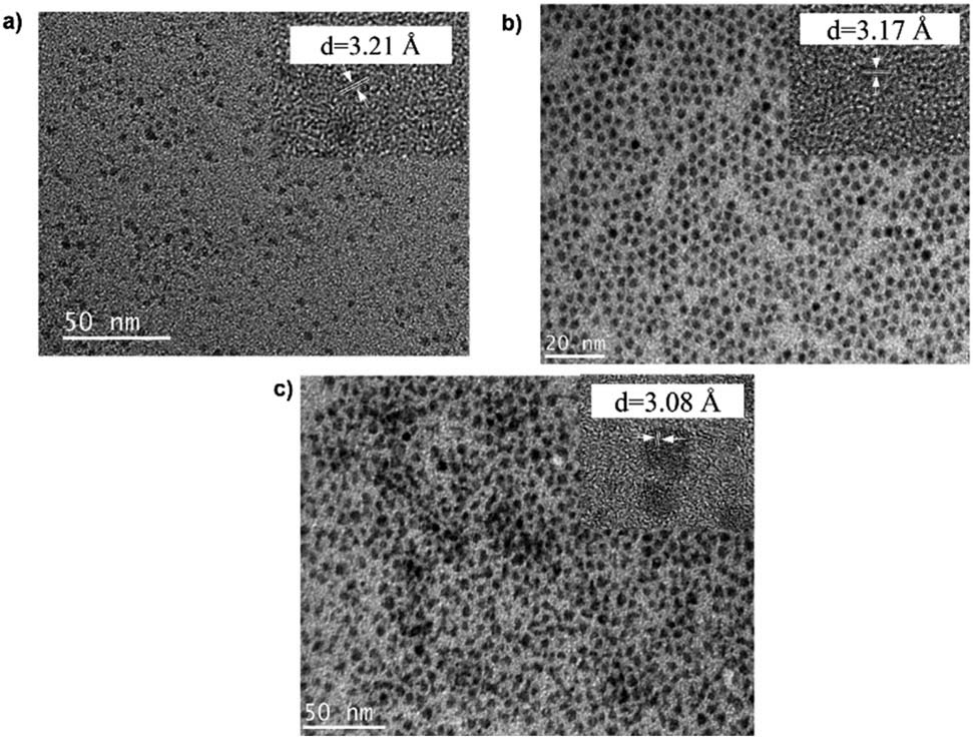
TEM images of CIS nanoparticles grown at (a) 140, (b) 180 and (c) 210 °C for 60 minutes with HR-TEM (inset) highlighting *d*_112_ interplanar spacing.

**Fig. 2:**
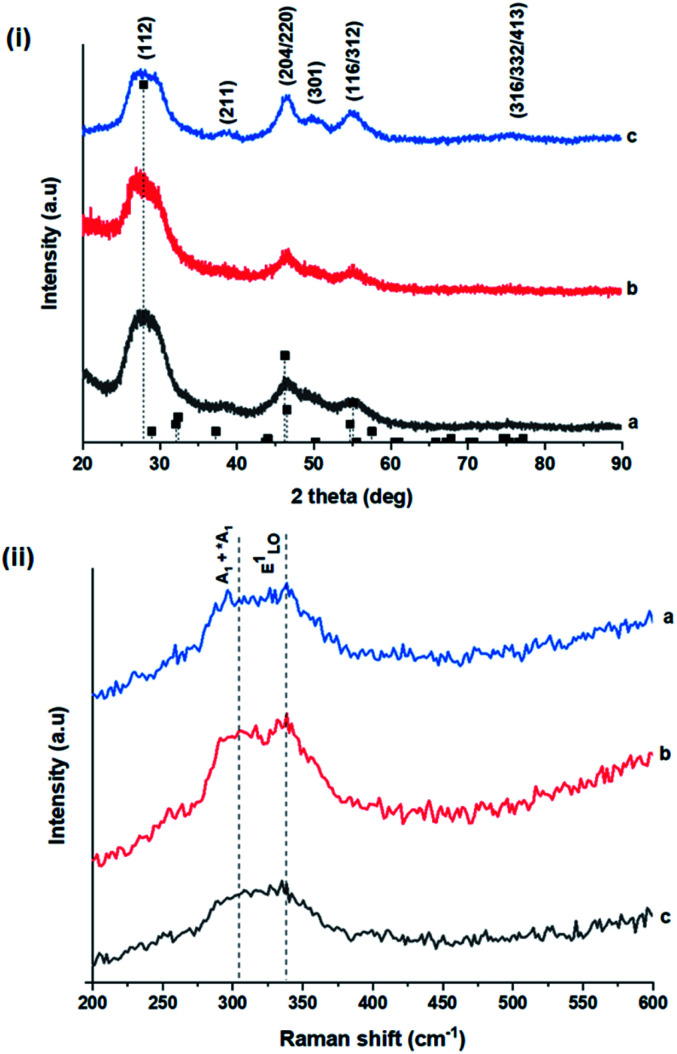
(i) Powder X-ray diffractograms of CIS nanoparticles grown at (a) 140, (b) 180 and (c) 210 °C for 60 minutes with reference peak positions for CIS chalcopyrite (tetragonal structure, ICDD reference no. 00-027-0159) shown as vertical dash lines and (ii) corresponding Raman spectra highlighting main vibrational modes.

TEM images in Fig. 1 show monodispersed (RSD = 13–18%; histograms in Fig. S2 ESI†) quasi-spherical nanoparticles of size 3.8–5.6 nm depending on growth temperature (Table 1). Inset HR-TEM images confirm the *d*_112_ lattice spacing of the CH structure (see Fig. S3 and Table S1 ESI†). Additionally, Fig. 2 shows overlaid p-XRD diffractograms of as-synthesized QDs prepared at respective temperatures with broad reflections typical of nanostructured particles, correlating well with the reference (tetragonal structure, ICDD reference no. 00-047-137) for the CH phase of CuInS_2_. Particle sizes calculated from the Scherrer formula^19^ compare well with estimates from HR-TEM, Table 1.

**Table 1:** Structural properties of CIS quantum dots evaluated from TEM, p-XRD and EDS measurements

Growth temperature/°C	Growth time/min	Particle diameter/nm		Elemental composition/%			Cu/In	Stoichiometry
		TEM (RSD/%)	PXRD^a^	Cu	In	S		
140	60	3.8 ± 0.7 (17.8)	3.0	19.0	23.5	56.6	0.8	Cu_0.80_In_0.94_S_2.26_
180	10	4.7 ± 0.5 (9.9)	4.0	23.5	26.0	50.5	0.9	Cu_0.94_In_1.04_S_2.02_
	60	5.2 ± 0.7 (12.9)	5.1	21.0	22.3	56.7	0.9	Cu_0.84_In_0.89_S_2.27_
210	60	5.6 ± 1.0 (17.2)	5.5	25.0	29.9	45.1	0.8	Cu_1.00_In_1.20_S_1.80_

a Calculated using Scherrer formula.^20^

As shown in Fig. 2, p-XRD data and Raman vibrational modes at approximately 474 and 323 cm^−1^ confirm phase pure QDs of no secondary binary phases and absence of the Cu_2_S and In_2_S_3_.^9,20^ The spectra display bands at around 295 and 305 cm^−1^, respectively related to S^2−^ ion A_1_ and *A_1_ vibrational modes which overlap to form a broad band.^21–23^ These along with the *E*^1^_LO_ band at 337 cm^−1^ suggest a mixed phase attributed to off-stoichiometry^21,24^ of CH and CA-type (copper–gold-type) tetragonal structures of a face centered cubic chalcogen lattice, differing in copper and indium sites. Since the CA-type phase is commonly associated with indium–copper anti-site 
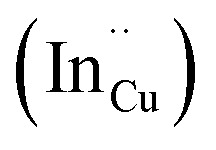
 defects^24,25^ and the ternary phase diagram^17^ of the Cu_2_S–In_2_S_3_ system suggests that copper-rich CIS typically possesses copper–indium anti-site 
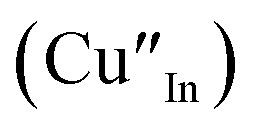
 and indium vacancy 
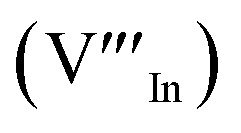
 defects, whilst indium-rich CIS possesses indium–copper anti-sites 
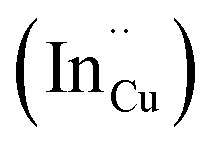
 and copper vacancies 
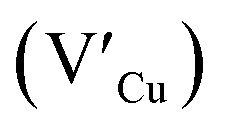
, we could on the basis of the defect chemistry attribute reasons for the structural and optical properties of the QDs. Notably, both 
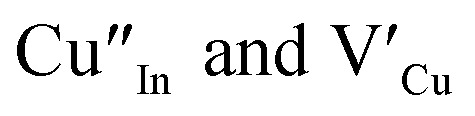
 produce p-type CIS with acceptor energy states slightly above the valence band maximum and sulphur-deficient CIS typically has sulphur vacancies 
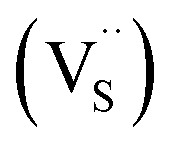
 of n-type conductivity with donor energy states slightly below the conduction band minimum.^26^

Additionally, in the bulk state, copper and sulphur-rich compositions influence transformation from metastable CA-type structure to the thermodynamically stable CH structure.^27^ Accordingly, on the premise of the relative intensity of the A_1_ and *A_1_ Raman vibrational modes and with reference to EDS elemental data (Table 1), the as-synthesized CIS quantum dots are copper-deficient, which possibly influences formation of an appreciable fraction of CA-type phase though the material is predominantly chalcopyrite.^21^ Since, annealing or extrinsic doping can possibly influence transition to the CH phase,^28^ we will report in a subsequent publication, the influence of Ag^+^ ion doping on phase transformation, the quality of the phase and tunable optical properties.

### Wurtzite to chalcopyrite phase transformation

**Fig. 3:**
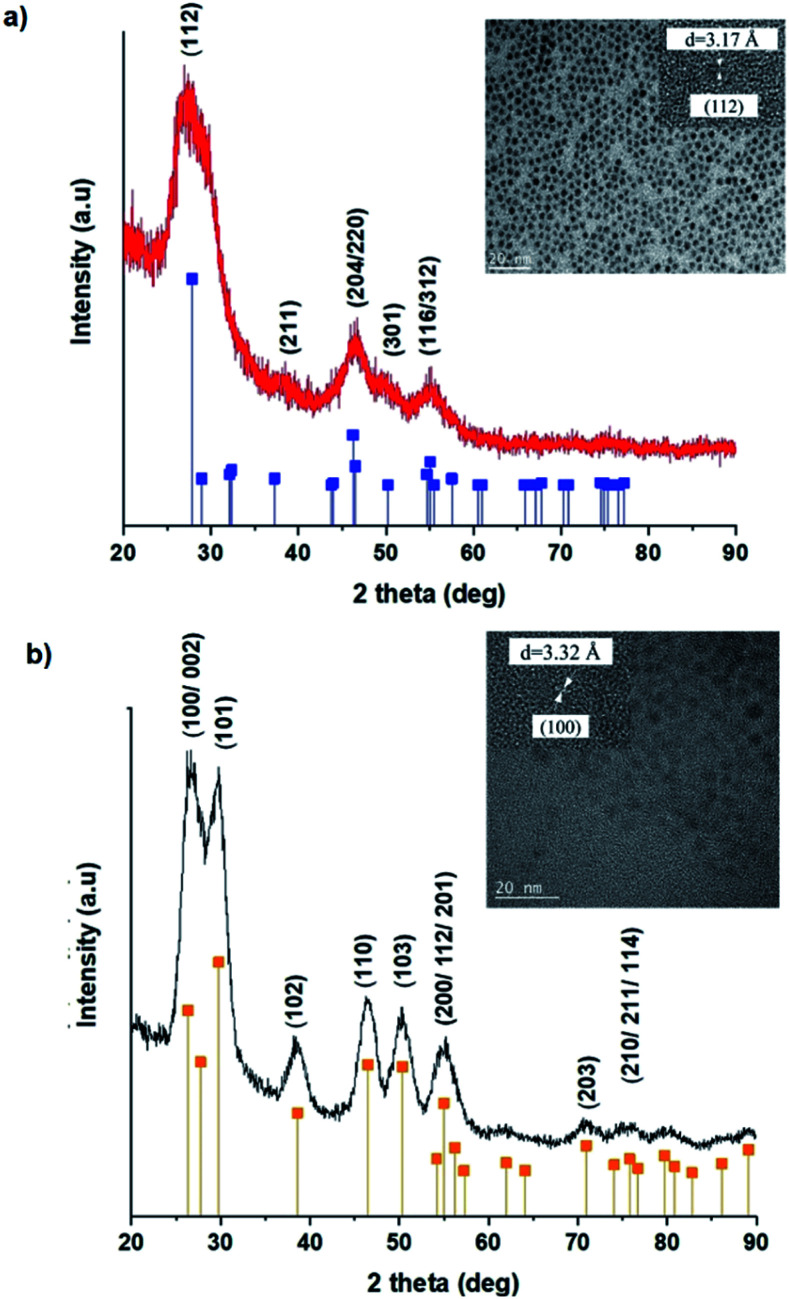
Powder X-ray diffractograms with inset showing HR-TEM with corresponding *d*-spacing for CIS nanoparticles grown at 180 °C for 60 minutes (top) with corresponding reference peak positions for CIS chalcopyrite (tetragonal structure, ICDD reference no. 00-027-0159) and CIS wurtzite grown at 180 °C for 10 minutes (bottom) (hexagonal structure, ICDD reference no. 01-077-9459) shown as vertical lines.

As previously stated, nanostructured CIS typically crystallizes as the stable CH, ZB or metastable WZ phases depending on synthetic factors.^18,29^ In our case, quantum dots grown at 180 °C for 10 minutes, unlike those grown for 60 minutes, possess a wurtzite structure as shown from X-ray diffractograms in Fig. 3, with characteristic reflections of (100)/(002) and (101) lattice planes at 2*θ* = 26.70° and 29.65°, respectively and suggests possible time-dependent structure selectivity. The inset highlighting the d-spacing of as-synthesized nanoparticles from HR-TEM corroborates this (see Table S1 ESI†). The WZ (hexagonal) phase may be linked to CH-CIS *via* a mechanism involving a dynamic interplay of kinetic and thermodynamic factors, further explored in subsequent discussions.

The thermodynamic stability of these chalcogenide phases was first documented in the pioneering work conducted by Binsma *et al.* and later concretized by Qi *et al.* in investigations which demonstrated the conversion of metastable WZ-CIS to the more thermodynamically favourable CH-CIS under elevated temperature.^30,31^ Though there are no reports of time-dependent WZ- to CH-CIS phase transformation at constant temperature, the conditions that influence the emergence of different chalcogenide phases are well established including the modulation of pH, solvent, reaction temperature as well as the nature and composition of the precursory material.^1,18,32–38^ Interestingly, work by Perera and team showed that wurtzite/chalcopyrite phase changes are not only thermodynamically driven as previously documented but can also be kinetically driven since reaction time, nucleation and subsequent growth rates were important factors in the crystal structure obtained.^36^ Chang *et al.* also demonstrated this through a series of kinetically controlled experiments where nanodisks and nanoparticles exhibited wurtzite/zinc blende phase selectivity.^18^

**Fig. 4:**
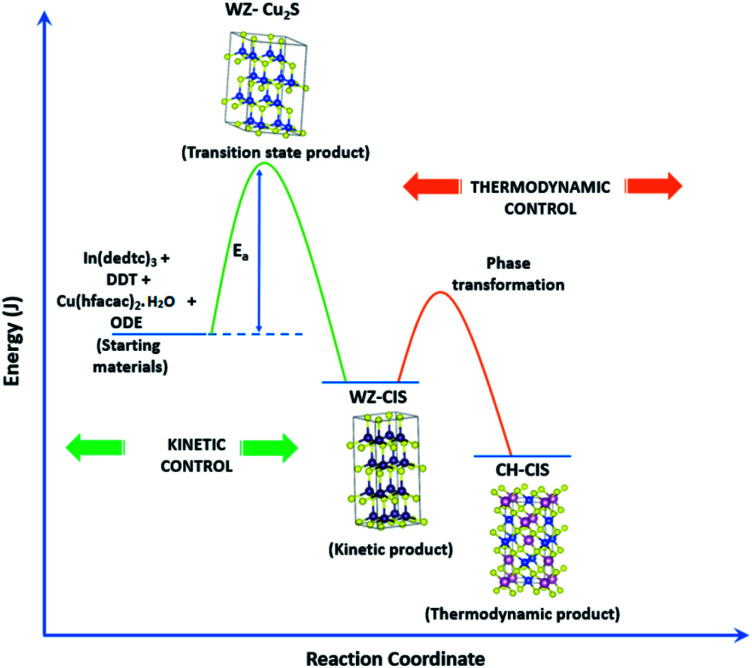
Schematic of proposed energy level profile illustrating the formation mechanism involving kinetic (WZ) and thermodynamic (CH) CIS.

Evaluation of these findings has enabled us to propose a particle growth mechanism illustrated in Fig. 4, in which the high growth rate in the early stages (up to 10 minutes) of synthesis involves a burst of nucleation upon precursor hot injection to form intermediate transition state Cu_2_S or Cu_2−*x*_S seeds and subsequent WZ CIS formation. It should be highlighted that Cu^+^ ions present in the binary chalcogenide are highly mobile and the abundance of vacancies within the binary copper chalcogenide structure accelerate cation exchange under kinetic conditions.^39,40^ As such, this drives the cation exchange of Cu^+^ and In^3+^ to form the kinetic product, metastable WZ CIS which subsequently undergoes transformation involving cation reordering to form the more thermodynamically stable phase at equilibrium when monomer concentration and growth rates decrease. Furthermore, kinetic factors are also dependent on the nature of the coordinating solvent.^3,35,41,42^

In our case, Cu_2_S/Cu_2−*x*_S seeds produce predominantly WZ phase nanoparticles since DDT as a soft Lewis base preferentially reacts with Cu^+^ ions (soft Lewis acid) than with hard In^3+^ ions as previously suggested. It is also reasonable to posit that polytypic wurtzite–chalcopyrite CIS nanostructures could intermediate the phase transition. Indeed, Koo *et al.* confirmed in predominantly wurtzite nanodisks, polytypism of wurtzite-chalcopyrite phases in which disks displayed wurtzite faces but chalcopyrite edges.^43^ Moreover from a structural standpoint, resultant CH-CIS phase from WZ-CIS is likely due to CH-(112) interface having a low lattice mismatch with WZ-(002) facet.^32,33,44^

In this regard, *a priori* assumption can be that defect formation mechanisms involving cationic reordering at the WZ–CH interface with low lattice mismatch to WZ CIS allow for the emergent thermodynamic chalcopyrite structure during particle growth. Though further experiments would allow for more in depth understanding of the kinetic and thermodynamic factors influencing such possible time-dependent phase transformation derived from this synthetic scheme, the current findings provide an alternative and facile route for phase selectivity not previously reported towards tunable properties of CIS nanoparticles.

### Absorption and photoluminescence properties

**Fig. 5:**
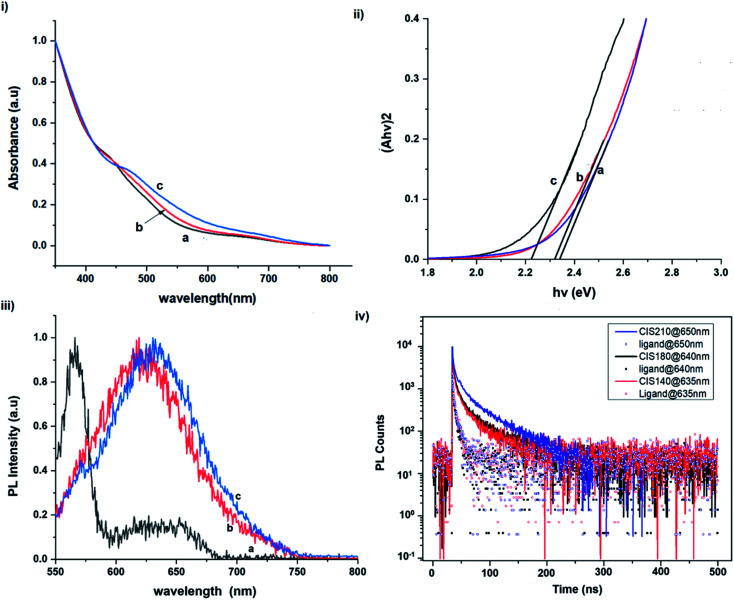
Room temperature normalized (i) absorption spectra, (ii) Tauc plots (*αhν*^2^*vs. hν*), (iii) photoluminescence spectra (at excitation wavelength of 480 nm) and (iv) corresponding decay curves (at excitation wavelength of 400 nm) of CIS nanoparticles grown at temperatures of (a) 140, (b) 180 and (c) 210 °C for 60 minutes relative to capping ligand.

The size, composition and phase-dependent optical tunability are exceptional features of CIS quantum dots to be investigated for quantum dot solar cells applications, for example. Accordingly, UV-vis spectra in Fig. S4 ESI† show a slight red-shift in band edge with particle growth time suggesting marginal size-dependence for the CIS quantum dots. However, corresponding photoluminescence spectra in Fig. S5 ESI† show a more distinct red-shift with the exception of quantum dots synthesized at 140 °C. The slight effect of growth temperatures 140, 180 and 210 °C, respectively are reflected in the direct band gaps of 2.32, 2.28 and 2.25 eV evaluated from Tauc plots shown in Fig. 5.^45,46^ Indeed, it has been established that the electronic band structure for CIS has a valence band comprised of Cu 3d and S 3p energy states from Cu–S bonds, whilst the conduction band is comprised of Cu 4s, S 3p and In 5s antibonding states.^47^ Since Laporte and spin-allowed direct band gap transitions are influenced by nanoparticle size, composition and structure,^18,48–51^ respective Cu/In ratio of 0.8, 0.9 and 0.8 from EDS analysis recorded in Table 1 confirm copper-deficiency which reduces the density of states associated with Cu 3d orbitals lowering the valence band maximum and widening the band gap. The extent of this effect is not clear since marginal particle size increase with growth temperature from 3.8 to 5.6 ± 0.7 nm is possibly a contributor.

Another factor for the shift in band gap may be the presence of surface defect states. Accordingly, nanoparticles synthesized at 140 °C exhibit photoemission characterized as a doublet peak at 568 and 630 nm of full-width-at-half-maximum (FWHM) = 19 and 67 nm respectively (see Fig. 5). For quantum dots synthesized at 180 and 210 °C, there is a slight-red shift in the emission maxima from 625 to 635 nm of FWHM = 98 and 91 nm, respectively with a shoulder at approximately 572 nm. The interesting, uncharacteristic doublet or shoulder features suggest competing mechanisms of electron–hole recombination. Though such broad emissions can be associated with broad size distributions^52^ (which is not so in this case), they are more likely associated with a combination of intra-band gap surface states and intrinsic defect states involving donor–acceptor pairs (DAPs) rather than band to band transitions.^3,51,53^ Since the quantum dots are copper-deficient, defects of 
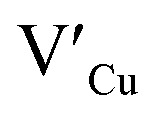
 as acceptors and donors of 
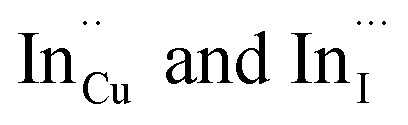
 are likely implicated as intrinsic intra-band gap states.^54,55^

**Fig. 6:**
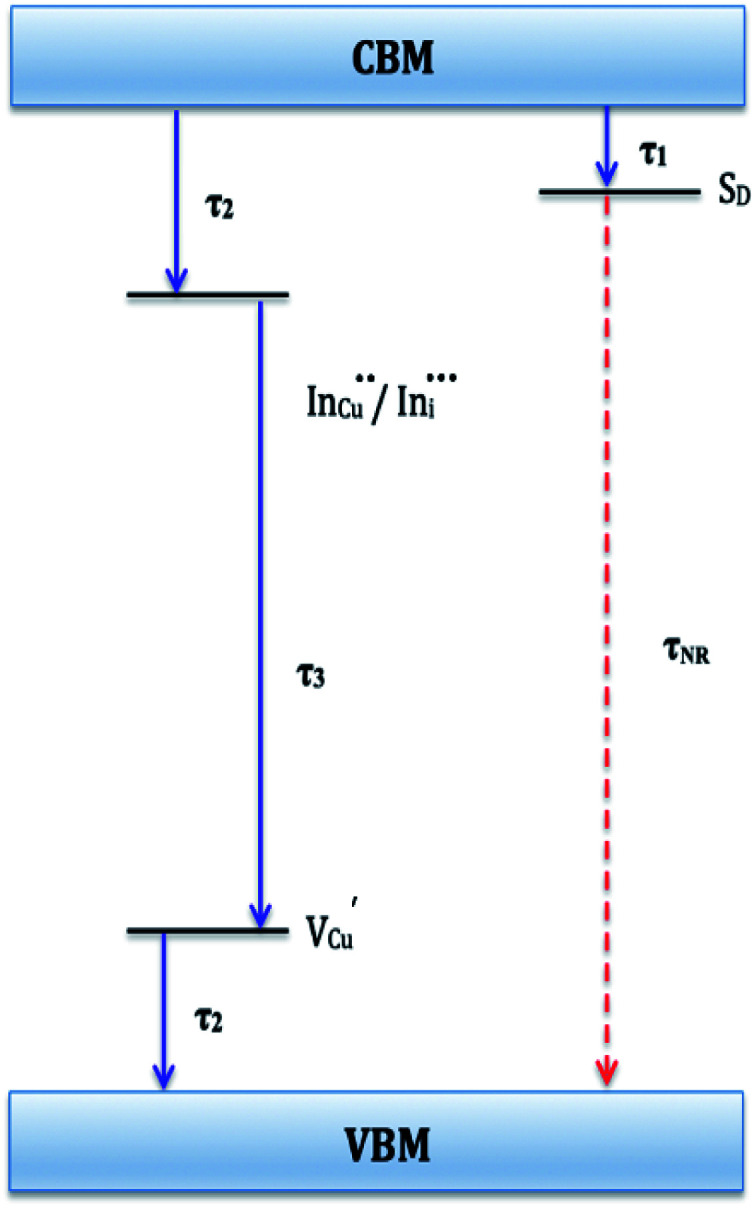
Schematic of proposed electronic band structure for CIS nanoparticles showing possible recombination pathways. *τ*_1_, *τ*_2_, and *τ*_3_ are radiative decay lifetimes and *τ*_NR_ is non-radiative decay lifetime.

Photoluminescence decay time measurements shown in Fig. 5 fit well to a tri-exponential function of the form:*I*_PL_(*t*) = *I*_0_ + *A*_1_e^−*t*/*τ*_1_^ + *A*_2_e^−*t*/*τ*_2_^ + *A*_3_e^−*t*/*τ*_3_^,with *τ*_1_, *τ*_2_, and *τ*_3_ being the lifetimes and *A*_1_, *A*_2_ and *A*_3_ the radiative components respectively. The *I*_0_ term is more likely related to the non-radiative recombination with a lifetime constant of *τ*_NR_ as illustrated in Fig. 6. The radiative lifetimes, *τ*_1_, *τ*_2_, and *τ*_3_ are found to be 1.50–2.1, 7.8–13.9 and 55.2–70.8 ns, respectively (Table 2). As illustrated in Fig. 6, the 1.5–2.1 ns decay is assigned to surface trapping states, *S*_D_ since these are usually shallow and therefore show faster decay lifetimes compared to intrinsic recombination of populated core states, which are assigned to 7.8–13.9 ns, respectively (Table 2).^8,56^ The longest lifetime, *τ*_3_ of 55.2–70.8 ns is likely related to internal defect states such as DAPs of 
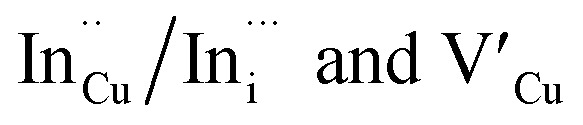
. Corresponding amplitude constants *A*_1_, *A*_2_ and *A*_3_ which range from 41.4–55.2, 38.4–49.8 and 6.4–14.0%, respectively indicate that the most dominant radiative recombination channels involve the faster decays between band edges and surface defects and 
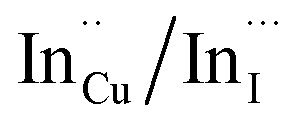
 with transition involving DAPs of 
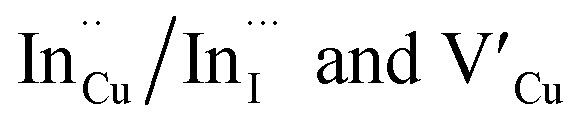
 contributing significantly less to the luminescence.

**Table 2:** Electronic absorption and emission properties of CIS quantum dots including radiative decay times (*τ*) with corresponding amplitude constants (*A*)

Growth temperature/°C	Growth time/min	Absorption		Emission		Radiative decay lifetime/ns and amplitude constant/%				
		*λ*_max_/nm	*E*_g_/eV	*λ*_max_/nm	*τ*_1_	*A*_1_	*τ*_2_	*A*_2_	*τ*_3_	*A*_3_
140	60	534	2.32	568^a^/630	1.5	55.2	10.3	38.4	55.2	6.4
180	10	610	2.03	656	—	—	—	—	—	—
	60	544	2.28	625^b^	1.3	41.4	7.8	49.8	54.5	8.8
210	60	551	2.25	635^b^	2.1	44.9	13.9	41.2	70.8	14.0

a Dominant.

b Shoulder at 572 nm; —not measured.

**Fig. 7:**
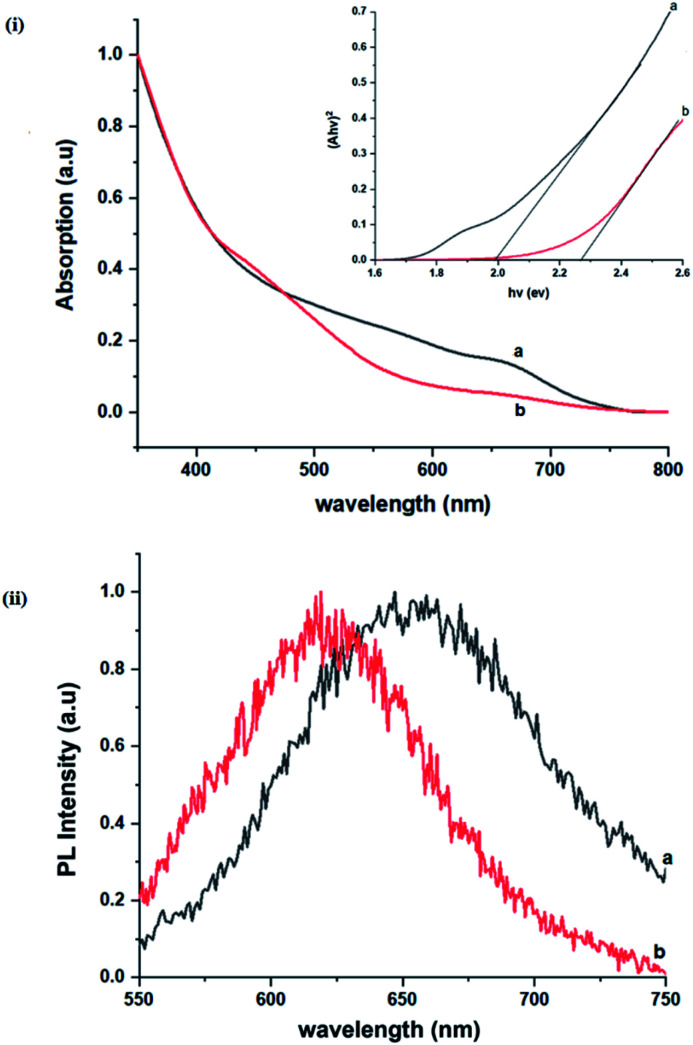
Room temperature (i) normalized absorption spectra and Tauc plots (*αhν*^2^*vs. hν*) (inset), (ii) corresponding normalized photoluminescence spectra (at excitation wavelength of 400 nm) of CIS nanoparticles grown at 180 °C for (a) 10 and (b) 60 minutes, respectively.

Importantly, there is a notable difference in optical properties between the WZ and CH phase CIS QDs. As shown in Fig. 7, the WZ phase CIS has a band gap of 2.03 eV which is red-shifted to that of the CH phase QDs at 2.28 eV and likewise the emission maxima (625–656 nm; CH–WZ). As shown in Table 1, with a marginal increase of approximately 0.5 nm and slight increase in the RSD of 3%, particle size variation is a minimal contributor. Certainly, it is difficult to ascertain the extent of the defects, 
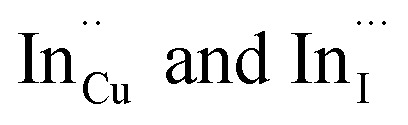
 arising from a higher concentration of In^3+^ ions for WZ CIS or the structural difference on the red-shift. In either case, the narrower band gap for the WZ phase CIS could be attributed to differences in the density of states arising from a higher concentration of states, primarily shallow defect states near the band edges.

## Conclusion

Relatively monodispersed colloidal CIS quantum dots (3.8–5.6 nm) of mixed phase chalcopyrite and copper–gold type structures were synthesized over a range of particle growth temperatures. The composition of nanoparticles synthesized at 140, 180 and 210 °C, respectively were very close to the CuInS_2_ stoichiometry with band gaps of 2.32, 2.28 and 2.25 eV, respectively. The copper-deficient quantum dots exhibit broad PL emission with a doublet/shoulder feature corresponding to decay lifetimes, *τ*_1_, *τ*_2_, and *τ*_3_ of 1.50–2.1, 7.8–13.9 and 55.2–70.8 ns respectively, indicate a combination of surface, donor and acceptor 
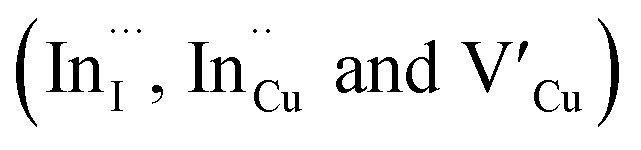
 defect states during electron–hole recombination. Notably, we were able to implicate wurtzite to chalcopyrite phase transformation at 180 °C, the first study of its kind involving possible time-dependent phase transformation at constant temperature, likely originating from a dynamic interplay of kinetic and thermodynamic factors influencing their phase. As such, this provided a unique route towards tunable structural and optical properties of colloidal CIS quantum dots.

## Experimental

### Materials

1-Dodecanethiol (DDT, Aldrich, >98%) and 1-octadecene (ODE, Aldrich, 90%) were used without further purification while methanol, acetone and toluene were distilled and dried using standard methods. Copper(ii) hexafluoroacetylacetonate hydrate [Cu(Hfacac)_2_·*x*H_2_O], sodium diethyldithiocarbamate trihydrate (Nadedtc·3H_2_O) and indium(iii) chloride (InCl_3_) were used as purchased from Aldrich. Indium(iii) diethyldithiocarbamate, In(dedtc)_3_ was prepared according to a literature method.^14^

### Synthesis of indium(iii) diethyldithiocarbamate precursor

Indium(iii) diethyldithiocarbamate, In(dedtc)_3_ was prepared according to a literature method.^14^ InCl_3_ (8.9 mmol, 1.9761 g) was dissolved in minimum amount of distilled water, cooled in an ice bath and then added dropwise to Nadedtc·3H_2_O (26.7 mmol, 6.0158 g), dissolved in 40 mL 50 : 50 mixture of methanol and water with constant stirring. In(dedtc)_3_ crystals were obtained by vacuum filtration and washed several times with methanol, recrystallized from toluene and air dried. Elemental analysis found (%): C, 33.02; H, 5.53; N, 7.39; S, 34.24; In, 20.47. Calculated for C_15_H_30_InN_3_S_6_ (559.63 g mol^−1^) (%): C, 32.21; H, 5.41; N, 7.51; S, 35.62; In, 20.53. IR (*ν*_max_, cm^−1^): 2864–2977(*ν*_s_ C–H), 1499 (*ν*_as_ C–N), 1268 (*ν*_as_ C–S), 986 (*ν*_s_ C–S).

### Synthesis and purification of DDT-capped CIS nanoparticles

In a routine synthesis, the first step involves vacuum then heating a mixture of In(dedtc)_3_ (0.2 mmol; 0.1169 g), DDT (2.4 mL) and ODE (5 mL) to 140 °C under argon. To this a hot mixture of Cu(hfacac)_2_·*x*H_2_O (0.2 mmol; 0.1038 g) and ODE (5 mL) was quickly injected followed by rapid heating to the respective particle growth temperature of either 140, 180 or 210 °C for a fixed time. Aliquots of as-synthesized nanoparticles at various time intervals were prepared from 0.5 mL of the reaction mixture and 1 mL of toluene for optical spectroscopy. As-synthesized nanoparticles were purified by reversible electrophoretic deposition adapted from Bass *et al.* using DC 50 V instead of 500 V removing the need for the use of a glovebox.^15^ Dispersed electropurified nanoparticles were centrifuged in acetone, isolated and later stored as solid or dispersed in toluene.

### Measurements

High-resolution transmission electron microscopy (HRTEM) was carried out using a FEI Talos F200X and Phillips 420 microscopes. For powder X-ray diffraction, nanoparticles as a toluene ink were drop-casted onto glass substrates, allowed to dry and mounted onto sample holders. Diffractograms were obtained using a Bruker D8 Advance X-ray diffractometer utilizing copper anode with filtered Cu-Kα radiation (*λ* = 0.15406 nm) within a 2*θ* range of 20 to 80° and step size of 0.02° s^−1^ for 2½ hours operating at 40 mA and 20 kV. Diffractograms were auto-indexed using the X'Pert HighScore software.^16^ Likewise, Raman spectra were recorded using a Renishaw RM system 1000 Mk1 Raman spectrometer with Modu-Laser Aries 163514/25 argon-ion 514 nm 25 mW laser at 0.277 mW with 50× objective lens within the range 26 to 1000 cm^−1^. Thermograms (Fig. S1 ESI†) of precursors were recorded between room temperature and 600 °C under nitrogen using a Mettler Toledo TGA/DSC1 instrument. CHNS data were recorded using a Thermo Scientific Flash 2000 elemental analyzer and metal ion contents measured using a Thermo Scientific iCAP 6300 Duo inductively coupled plasma-optical emission spectrometer (ICP-OES). Energy dispersive spectroscopic (EDS) data on nanoparticles were recorded in scanning electron microscopy (SEM) mode using a Phillips XL 30 field emission gun (FEG) microscope with electron beam accelerating voltage of 20 kV and working distance of 10 mm. Optical spectroscopy of nanoparticles dispersed in toluene was possible using a Perkin Elmer Lambda 1050 UV/vis/NIR spectrometer within the range of 350 to 800 nm and a Perkin Elmer LS55 fluorescence spectrofluorometer with excitation wavelength of 480 nm within the region of 550 to 880 nm. Photoluminescence time decay measurements were performed using a time-correlated-single-photon-counting setup. The output from a mode-locked Ti:sapphire laser (Mai Tai HP, Spectra-Physics), which provided 100 fs pulses at a repetition rate of 80 MHz and a central wavelength of 820 nm, was frequency doubled to 410 nm to provide the required wavelength for photo-excitation. An acousto-optic pulse picker (Pulse Select, APE) was employed to reduce the repetition rate of the laser pulse train to 2 MHz, and thereby increase the measurement time window. After photo-excitation the PL emission was directed into a monochromator (Spex 1870c) and detected at the PL peak (or tuned at the preferred wavelength within the range of the PL spectra of each sample) by a multi-channel plate (Hamamatsu R3809U-50). The time correlation of the detected photons was performed using a PC card (TCC900, Edinburgh Instruments).

## Conflicts of interest

There are no conflicts to declare.

## Supplementary Material

RA-011-D1RA03659A-s001
