# Supplementary material for: Tunable structural and optical properties of CuInS2 colloidal quantum dots as photovoltaic absorbers
Source: RSC Adv. 2021 Jun 16;11(35):21351–8. doi: 10.1039/d1ra03659a (PMC9034010; doi:10.1039/d1ra03659a)
Supplement: RA-011-D1RA03659A-s001 [file RA-011-D1RA03659A-s001.pdf]

Electronic Supplementary Material (ESI) for RSC Advances.
This journal is © The Royal Society of Chemistry 2021

Electronic Supplementary Information (ESI)

Tunable Structural and Optical Properties of $CuInS_2$ Colloidal Quantum Dots as Photovoltaic Absorbers

Shanna-Kay Ming [a], Richard A. Taylor*[a], Paul D. McNaughter [b], David J. Lewis [c] Marina A. Leontiadou [d,e] and Paul O'Brien [b,c],

[a]Department of Chemistry, University of the West Indies, St. Augustine, Trinidad and Tobago.
[b]Department of Chemistry, University of Manchester M13 BB, United Kingdom
[c]Department of Materials, University of Manchester, Manchester M13 9PL , United Kingdom.
[d]Department of Physics and Astronomy & Photon Science Institute, The University of Manchester, Manchester M13 9PL, United Kingdom.
[e]Present Address: Joule Physics Laboratory, School of Science, Engineering & Environment, University of Salford, Manchester M5 4WT, United Kingdom.
*Corresponding author E-mail: richard.taylor@sta.uwi.edu

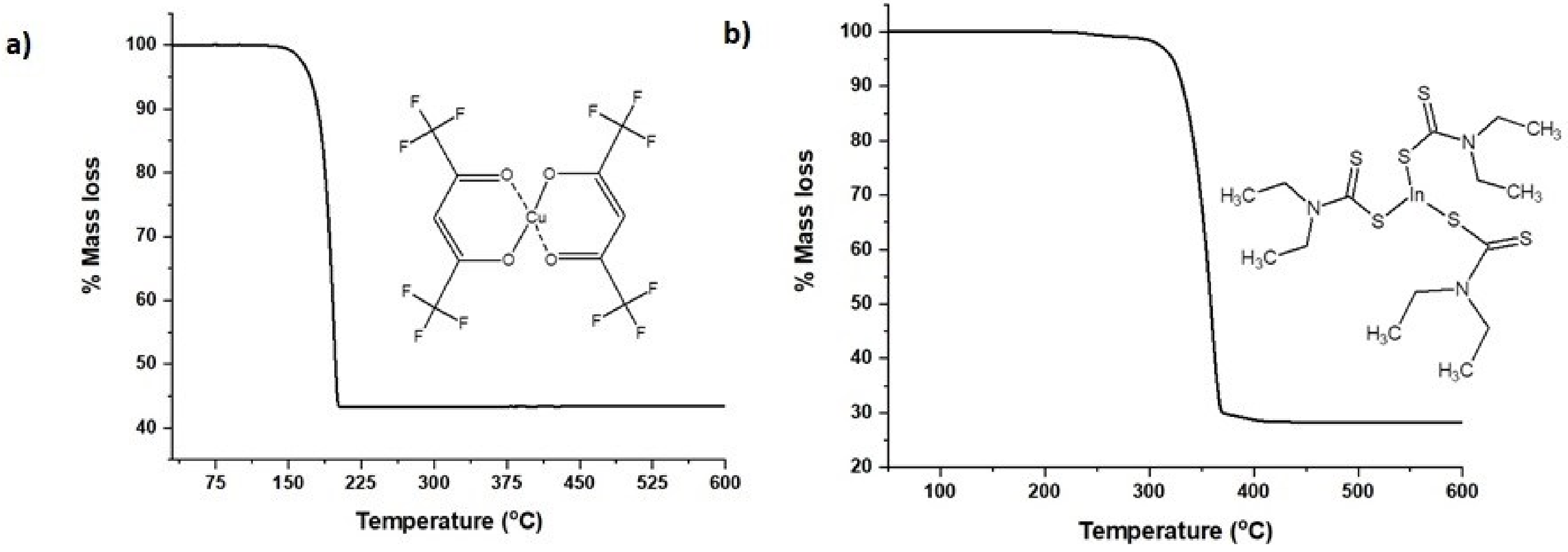

Figure S1: TGA thermograms for a) $Cu(Hfacac)_2.xH_2O$ b) $In(dedtc)_3$ with respective molecular structures.

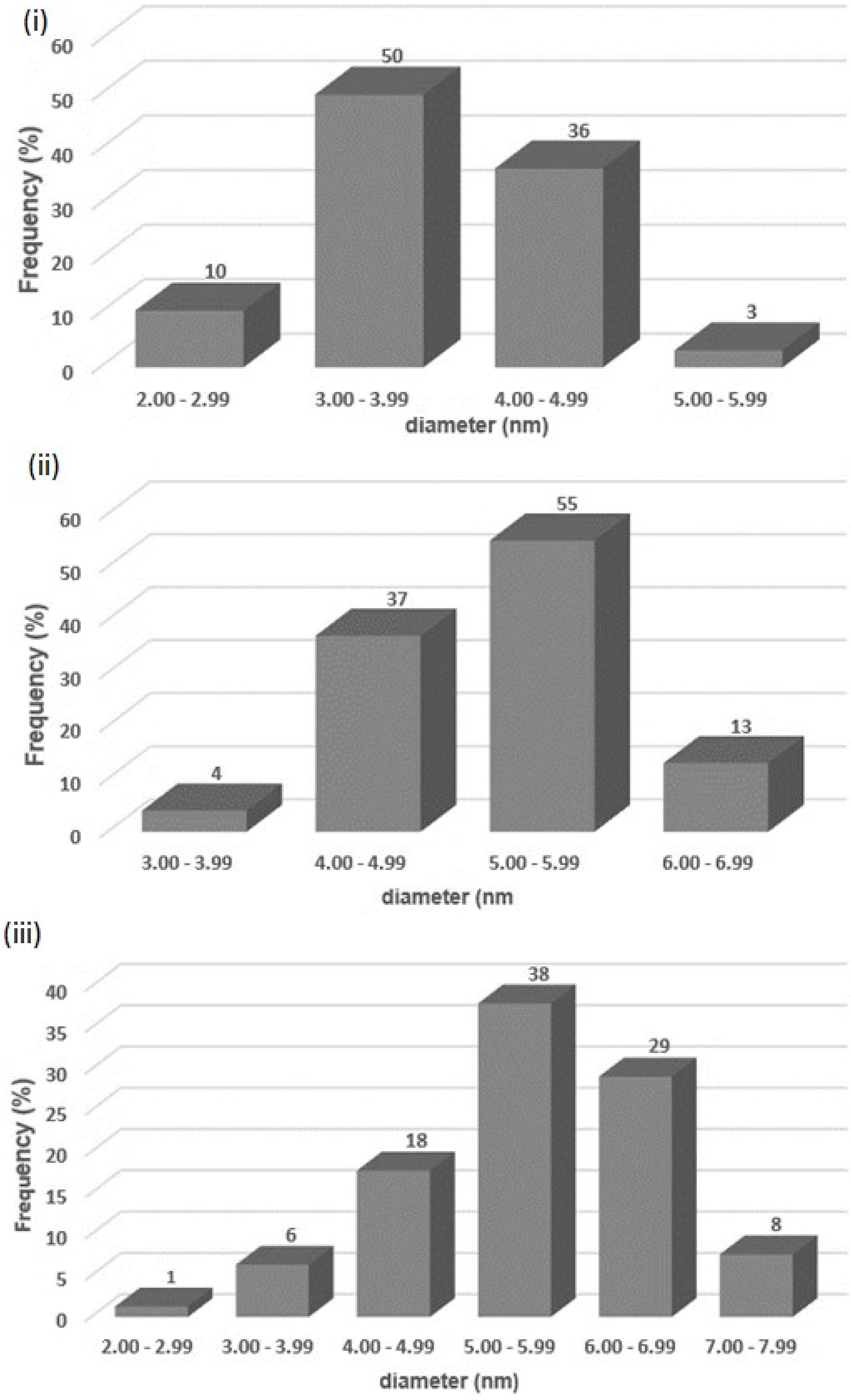

Figure S2: Histograms showing size distribution from TEM of CIS nanoparticles synthesized at (i) 140, (ii) 180 and (iii) 210 ^{o}C, respectively for 60 minutes.

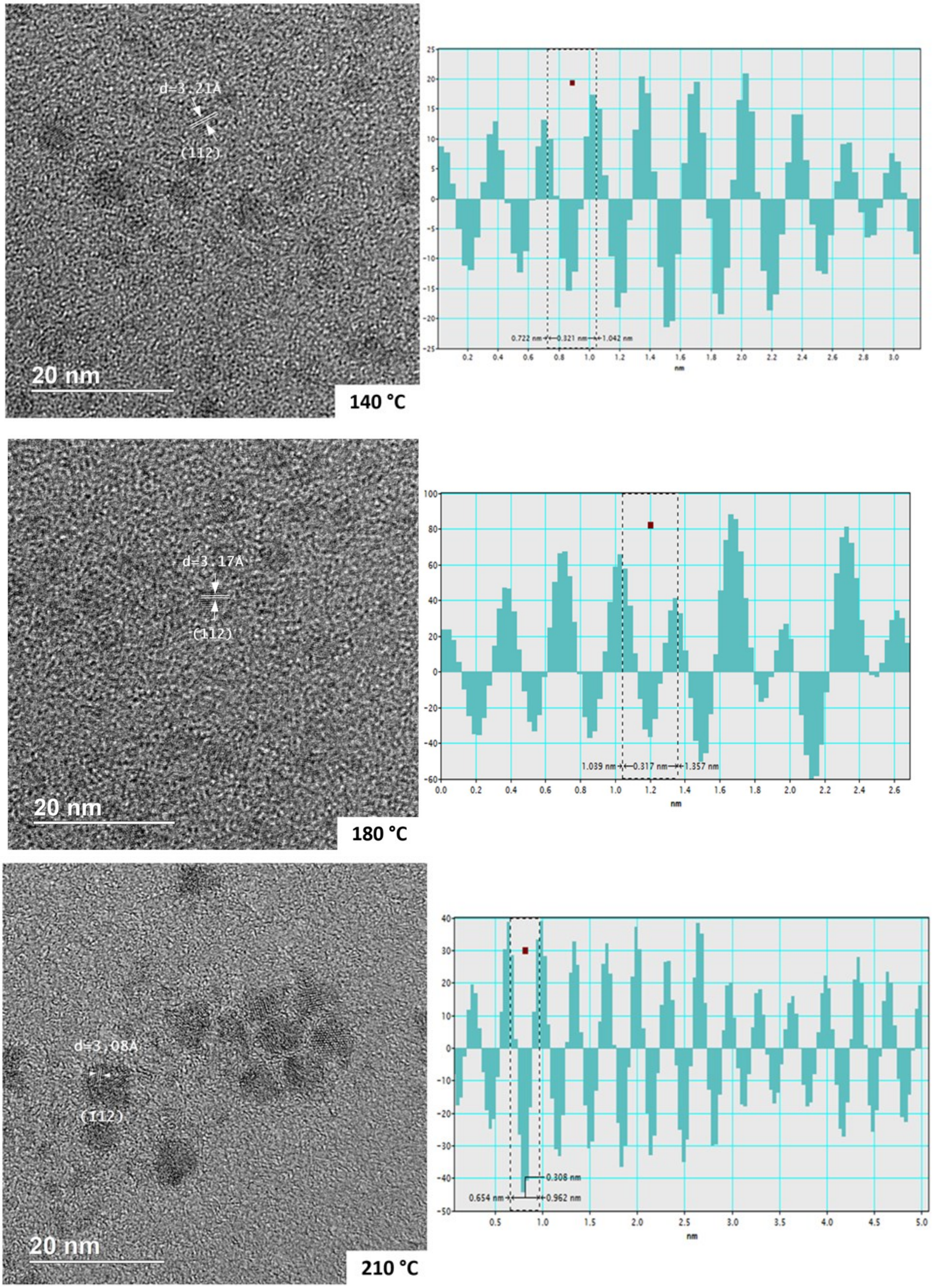

Figure S3: (Left) HR-TEM images of nanoparticle ensemble at different reaction temperatures with inset highlighting lattice spacings (right) corresponding lattice spacing measurements of 3.21, 3.17 and 3.08 Å.

Table S1: Lattice parameters, hkl values and corresponding d-spacings of synthesized chalcopyrite and wurtzite nanoparticles.

Crystal Lattice: Tetragonal<br>Space Group I-42d<br>a = b = 5.5230, c = 11.14<br>α = β = γ= 90°		
hkl	d-spacing	2theta
112	3.22541	27.634
204/220	1.94654	46.6228
116/312	1.65718	55.3979
Crystal Lattice: Hexagonal<br>Space Group P63mc<br>a = b= 3.9065, c = 6.4290<br>α =β = 90° and γ= 120°		
hkl	d-spacing	2theta
100/002	3.32337	26.8041
101	3.00959	29.6595
102	2.31923	38.7969
110	1.95491	46.4113
103	1.81526	50.2186
112	1.66885	54.9776
203	1.33012	70.7776
211	1.255	75.727

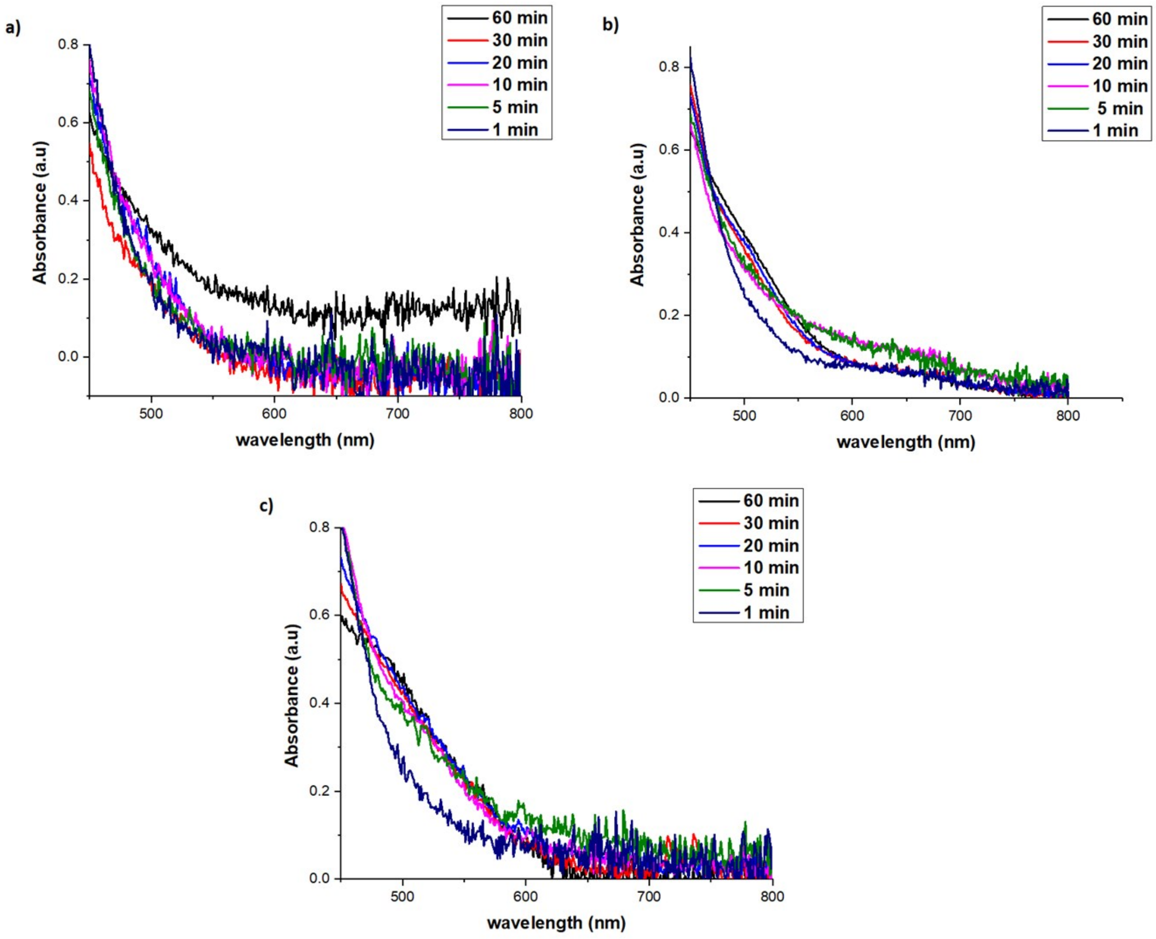

Figure S4: UV-vis absorption spectra of aliquots taken at specific intervals from reactions at temperatures a) 140 b) 180 c) 210 ^{o}C, respectively.

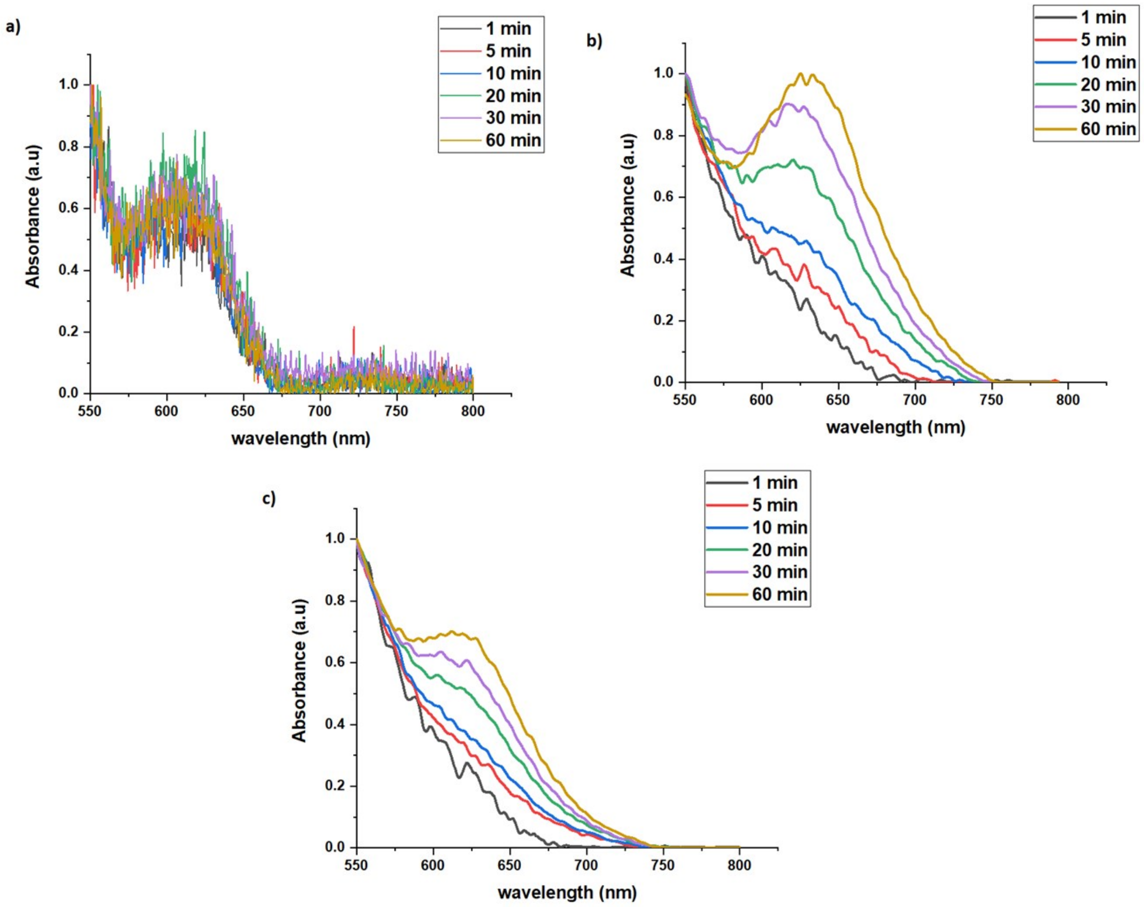

Figure S5: Photoluminescence spectra of aliquots taken at specific intervals from reactions at temperatures a) 140 b) 180 c) 210 ^{o}C (λ_{exc} = 440 nm).
